# Skimmianine Attenuates Osteoclast Activity by Suppressing ERp57‐Driven Calcium Oscillations/Calcineurin/Nfatc1 Signalling in Postmenopausal Osteoporosis

**DOI:** 10.1111/jcmm.70777

**Published:** 2025-08-12

**Authors:** Yongshuang Lv, Xin Zhang, Qizhen Lu, Yi Zhou, Weiyi Wang, Maosheng Yang, Tao Yuan, Yikai Liu, Shui Sun, Ziqing Li

**Affiliations:** ^1^ Department of Joint Surgery Shandong Provincial Hospital Affiliated to Shandong First Medical University Jinan Shandong China; ^2^ Orthopaedic Research Laboratory, Medical Science and Technology Innovation Center Shandong First Medical University & Shandong Academy of Medical Sciences Jinan Shandong China; ^3^ Wenzhou Hospital of Traditional Chinese Medicine Affiliated Zhejiang University of Traditional Chinese Medicine Wenzhou China; ^4^ Department of Joint Surgery, Shandong Provincial Hospital, Cheeloo College of Medicine Shandong University Jinan Shandong China; ^5^ The Second Clinical Medical College of Shandong University of Traditional Chinese Medicine Jinan Shandong China

**Keywords:** Calcium oscillations, Calcium signalling, Osteoclast, Postmenopausal osteoporosis, Protein disulfide isomerase A3 (PDIA3/ERp57), Skimmianine (Ski)

## Abstract

Postmenopausal osteoporosis is primarily attributed to the hyperactivation of osteoclast‐induced bone resorption. The differentiation and function of osteoclasts rely on the regulation of calcium oscillations/calcineurin/nuclear factor of activated T cells (Nfat) pathway. Therefore, the development of natural compounds that aim at the crucial regulator of the aforementioned pathway is essential for the suppression of osteoclastogenesis and its clinical application. Skimmianine (Ski), a furoquinoline alkaloid extracted from the Zanthoxylum genus, is known for its anti‐inflammatory properties. Yet, its exact role in osteoclast differentiation and function remains largely undefined. Herein, we evaluate the impact of Ski on osteoclastogenesis and elucidate the molecular mechanism involved. We conducted network pharmacology and molecule structure‐based pharmacokinetics analyses on Ski, followed by experimental validations on osteoclastogenesis in vitro and ovariectomy (OVX) mice model in vivo. The network pharmacology results indicated Ski's therapeutic effects predominantly influence the calcium signalling pathway by controlling cytosolic calcium concentration in response to bone resorption during osteoporosis. Pharmacokinetic analyses revealed Ski's excellent oral bioavailability. Furthermore, experimental validations revealed that Ski inhibited the formation of multinucleated osteoclasts in a concentration‐dependent manner without affecting cell viability, while impeding osteoclast‐related gene expression. The underlying mechanism involved the Ski‐induced downregulation of calcineurin/Nfatc1 expression through modulation of ERp57‐driven calcium oscillations. Micro‐CT results confirmed that Ski treatment substantially curbed the progression of osteoporosis by mitigating bone loss. In conclusion, our findings indicated that Ski suppressed osteoclast formation by suppressing ERp57‐driven calcium oscillations/calcineurin/Nfatc1 signalling, thus establishing Ski as a promising therapeutic alternative for osteoporosis.

## 1 Introduction

Osteoporosis is a systemic disease marked by the reduction of bone mineral density (BMD) and deterioration of bone tissue microarchitecture, resulting in bone fragility and increased fracture risk [1]. According to recent reports, women aged 55 and above, along with men aged 65 and above, endure an increased risk of fractures consequential to osteoporosis [2]. Despite not having a complete understanding of the exact mechanisms involved, postmenopausal osteoporosis is characterised by an imbalance in bone remodelling caused by oestrogen deficiency. In particular, the excessive bone‐resorbing activity of osteoclasts is primarily responsible for the pathological degradation of bone tissue [3, 4]. Indeed, while anti‐osteoporotic treatments such as denosumab and bisphosphonates are commonly used to target osteoclasts, they do carry a risk of jaw necrosis and atypical femur fracture, a condition that can lead to severe disability [5, 6, 7]. Thus, promising alternative therapeutic agents for osteoporosis that can target osteoclastogenesis through different mechanisms with improved risk–benefit profiles are urgently needed.

Osteoclasts, arising from the fusion of mononuclear progenitor cells, are giant polykaryotic cells that originate from the bone marrow haematopoietic stem cell lineage and are responsible for bone resorption [8]. Similarly, the rate of bone resorption relies on the lifecycle of osteoclasts, which is governed by the expression of osteoclast‐specific marker genes such as TNF receptor‐associated factor 6 (Traf6) and nuclear factor of activated T‐cells 1 (Nfatc1) [9], followed by the expression of tartrate‐resistant acid phosphatase (TRAP) and the secretion of acid and collagenase such as cathepsin K (Ctsk), ultimately resulting in the degradation of bone matrix [8, 10]. Previous studies have clearly confirmed that protein disulfide isomerase A3 (PDIA3/ERp57) plays a crucial role in the calcium signal transduction process [11]. Calcium oscillations, characterised by periodic variations in intracellular calcium levels that activate calcium signalling pathways, have been experimentally confirmed as critical regulators of osteoclast development and activity [12, 13]. As the downstream effector, calcineurin not only plays a critical role in calcium signalling within osteoclasts, but also serves as the primary regulator of Nfatc1 activation. Upon receiving extracellular stimuli, the entry of calcium into the cytosol triggers calcineurin activation. Activated calcineurin then facilitates signal transmission from the cytosol to the nucleus by dephosphorylating the transcription factor Nfatc1 and initiating its nuclear translocation [14], followed by complex formation between Nfatc1 and other transcription factors. This ultimately induces the expression of osteoclast marker genes including Ctsk, matrix metallopeptidase 9 (MMP9) and the d2 isoform of vacuolar (H+) ATPase Vo domain (Atp6v0d2), driving hyperactivation of osteoclasts and increased bone erosion [15, 16]. As such, inhibiting osteoclast hyperactivation would be an effective therapeutic strategy for postmenopausal osteoporosis via the correction of calcium oscillations‐calcineurin‐Nfatc1 pathway‐regulated bone remodelling imbalance.

Traditional Chinese medicines (TCMs) offer several advantages in treating osteoporosis, including effectiveness, safety and multi‐directional action. Integrating TCMs into osteoporosis treatment provides a dual approach, addressing both symptoms and root causes by promoting microcirculation, and exerting anti‐inflammatory and analgesic effects [17]. The fruit of Zanthoxylum piperitum (Rutaceae) has been used as food and herbal medicine for treating carminative, stomachic and anthelmintic disorders in East Asia [18]. Previous studies confirmed its anti‐inflammatory, anti‐osteoarthritic and anti‐osteoporotic properties [19, 20]. Skimmianine (Ski) is the most abundant furoquinoline alkaloid found in the Zanthoxylum genus and other Rutaceae genera [21, 22, 23], with reported anti‐inflammatory effects [24]. Despite bone marrow macrophages and monocytes are potential osteoclast precursors, it remains unclear whether Ski regulates osteoclast differentiation or function. Bioinformatics advancements have enabled network pharmacology, a state‐of‐the‐art technique, that updates the ‘one target, one drug’ paradigm through intricate drug‐target‐pathway networks [25].

Hence, in the present study, we used network pharmacology databases to establish Ski's pharmacological network for osteoporosis and predict potential gene targets and pathways. Subsequent in vitro experimental verification provided definitive evidence of Ski's suppression on osteoclasts via the calcineurin–Nfatc1 signalling pathway, accompanied by downregulated expression of osteoclast differentiation factors (Nfatc1, Ctsk, MMP9 and Atp6v0d2). These findings identify Ski as a novel candidate for ameliorating bone loss and provide a theoretical basis for developing Ski as an anti‐osteoporosis drug.

## 2 Methods

### 2.1 Network Analysis of Overlapping Targets of Ski in Osteoporosis

**FIGURE 1:**
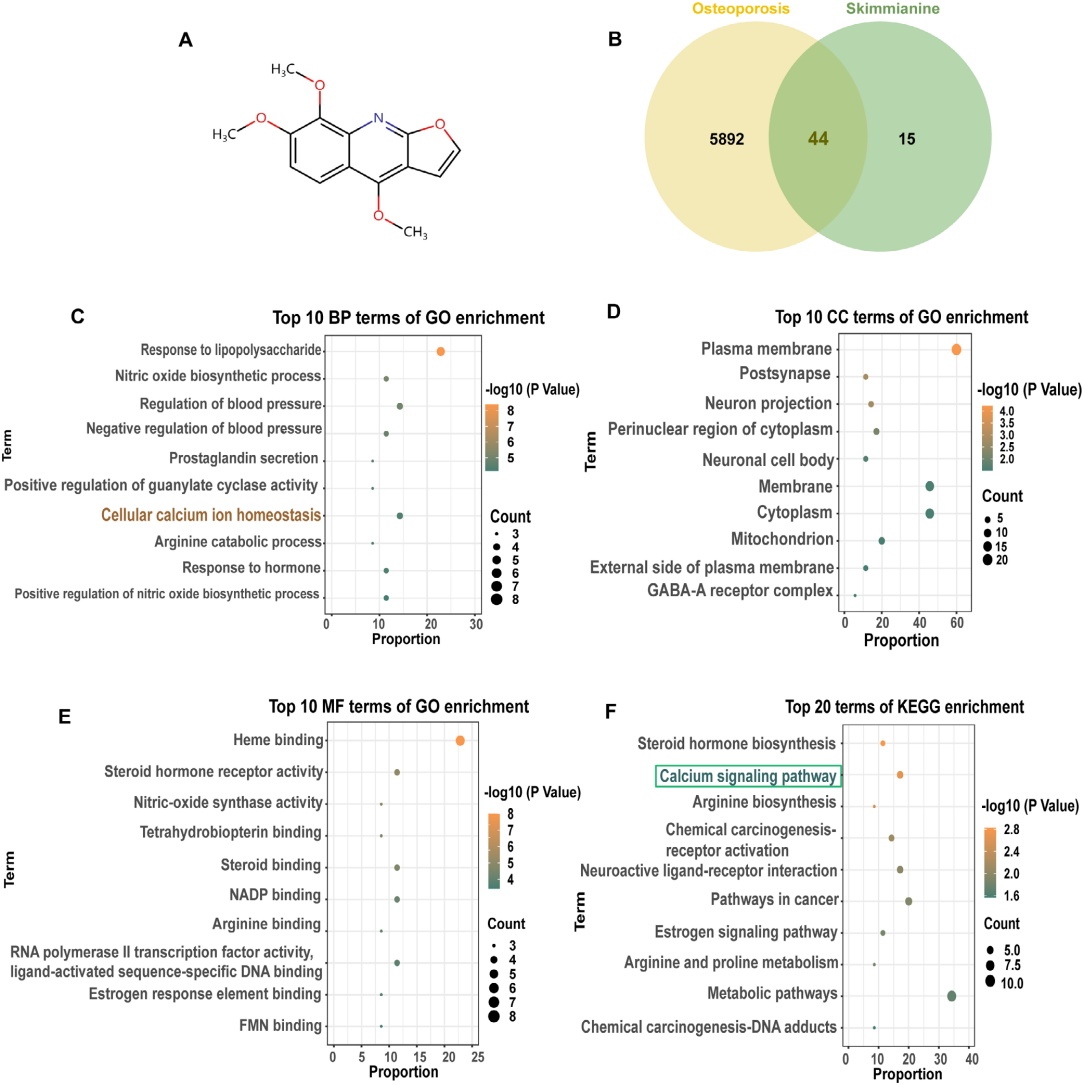
The prediction of the biological pathway of Ski in osteoporosis prevention and treatment. (A) Molecule structure of Ski. (B) Venn diagram exhibited the number of putative targets of Ski on osteoporosis. (C–E) GO enrichment analysis of Ski, including the top 10 terms of biological process (BP), molecular function (MF) and cell component (CC), consistent with the candidate targets. (F) KEGG pathway analysis for identification of the tightly interrelated association between Ski and calcium signalling pathway.

The predicted targets with relevance greater than 0 were collected from the Swiss Target Prediction database (http://www.swisstargetprediction.ch/) [26] pursuant to the structure of Ski (Figure 1A). According to the absorption, distribution, metabolism and excretion (ADME) characteristics of the drugs in the body, Analysis Platform (TCMSP, https://tcmsp‐e.com/) [27] was used for identifying Ski with oral bioavailability (OB) ≥ 30% [28] and drug‐likeness (DL) ≥ 0.18 [29] as compounds with pharmacological activity. Genes responsible for osteoporosis‐associated pathogenesis were acquired from the GeneCards database (https://www.genecards.org/) using ‘osteoporosis’ as a keyword. The potential targets of Ski subsequently were sustained to map with the targets related to osteoporosis in order to acquire common targets. The Venn diagram website (https://jvenn.toulouse.inrae.fr/app/example.html) [30] was used to obtain a Venn diagram to identify the overlapping genes between Ski and osteoporosis. The online database DAVID Bioinformatics Resources 6.8 (DAVID) (https://david.ncifcrf.gov/home.jsp) [31] was employed to study Gene Ontology (GO) and Kyoto Encyclopedia of Genes and Genomes (KEGG) pathway enrichment for regulated genes of Ski and osteoporosis. (The time of the databases were based on January 2024.).

### 2.2 Pharmacokinetics Studies and ADME Properties (Absorption, Distribution, Metabolism and Excretion)

The Lipinski Rule of Five helps differentiate between drug‐like and non‐drug‐like properties [32]. To predict Ski's physicochemical and pharmacokinetic characteristics, including ADME parameters and medicinal chemistry features, the SwissADME tool (http://www.swissadme.ch/) was employed [33]. The bioavailability radar assesses Ski's drug‐likeness by evaluating six key properties: lipophilicity (LIPO), size (SIZE), polarity (POLAR), insolubility (INSOLU), insaturation (INSATU) and flexibility (FLEX) (Table S1). For estimating brain penetration and intestinal absorption, the BOILED‐Egg model was utilised. This intuitive graph correlates lipophilicity (WLOGP) with permeability (TPSA) [34].

### 2.3 Molecular Docking Simulation

The molecular dockings of Ski, ERp57 and calcineurin protein were performed to corroborate their potential interaction activity and explore the accurate binding mode. The SDF format file of Ski was acquired from the PubChem database (https://pubchem.ncbi.nlm.nih.gov/) and the protein structures of ERp57 and calcineurin were fetched from the Protein Data Bank (https://www.rcsb.org). The above files were subjected to pre‐processing steps, including the removal of water molecules, separation of ligands and addition of hydrogen atoms. The pair of protein‐ligand complex systems with the strongest binding capacity from 100 combination situations was selected for visualisation using the MOE software (2022.02).

### 2.4 Reagents and Antibodies

Reagents with regard to cell culture, for instance, fetal bovine serum (FBS, 10099141C), minimum essential medium α (α‐MEM, C12571500BT) and penicillin/streptomycin (P/S, 15140122), were acquired from Gibco (USA). Primary antibodies subjected to western blotting (WB) for the detection of Ctsk (sc‐48353), PP2B‐Aα (sc‐17808) and β‐actin (sc‐47778) were acquired from Santa Cruz; primary antibodies for Nfact1 (A1539) detection were obtained from ABclonal Technology. All secondary antibodies conducted on experiments, containing anti‐rabbit (SA00001‐2) and anti‐mouse (SA00001‐1) were acquired from Proteintech. Other biochemistry reagents for cell stimulation and differentiation included Skimmianine (Ski, 83‐95‐4, MCE), dimethyl sulphoxide (DMSO, D2650, Sigma‐Aldrich), macrophage colony‐stimulating factor (M‐CSF, 576406; BioLegend) and receptor activator of nuclear factor‐κB (RANK) ligand (RANKL, 462‐TEC‐010, R&D Systems). TRAP staining reagents include sodium tartrate dibasic dihydrate (6106‐24‐7, Sigma‐Aldrich), sodium acetate trihydrate (6131‐90‐4, Sigma‐Aldrich), Fast Red Violet LB (32348‐81‐5, In vivoChem), glacial acetic acid (100%) (1000631011, Sigma‐Aldrich), naphthol AS‐TR phosphate disodium salt (4264‐93‐1, Sigma‐Aldrich) and sodium fluoride (HY‐B1766, MCE). The fluorescent probe Fluo‐4 (20551) was acquired from AAT Bioquest. Isotonic solution (ISO) materials were obtained from Solarbio, including NaCl (WY‐SLB‐S8210‐500), KCl (P9921), HEPES acid (WY‐SLB‐H8090‐25), Na HEPES (H9020‐25) NaHCO_3_ (S5240‐500), D‐mannitol (WY‐SLB‐M8140‐250), D‐glucose (WY‐SLB‐G8150‐250), CaCl_2_ (C7250) and MgCl_2_ (M8161).

### 2.5 Cell Culture and Osteoclast Differentiation

Bone marrow‐derived monocytes (BMMs) were obtained from 8‐ to 12‐week‐old C57BL/6 male mice according to our previous study [35]. After being flushed out from the femur and tibia, bone marrow cells were developed in a complete medium (α‐MEM, 10% FBS and 1% P/S) at 37°C in a 5% CO_2_ atmosphere for 16–18 h. The non‐adherent cells were taken out and then processed with red blood cell lysate (BL503A, Biosharp) for 3 mins. Bone marrow‐derived monocytes/macrophages (BMMs) were cultured in a complete medium containing M‐CSF (10 ng/mL) at a density of 1.5 × 10^5^/mL for 48 h and then induced with the osteoclast induction medium (OIM) containing M‐CSF (10 ng/mL) and RANKL (30 ng/mL) for 1–3 days to generate osteoclast precursor cells or 4–5 days to generate functionally mature osteoclasts, which were defined as the control group. Ski was dissolved in DMSO to generate a storage solution (30 mM) and then diluted to different working concentrations (30–45 μM) by using α‐MEM. In terms of treatment, groups were given Ski at indicated concentrations. Culture medium change was performed after 48 h of the first RANKL addition and then changed daily until cell harvest.

### 2.6 Cell Viability Assay

The cytotoxic effects of Ski on osteoclast precursor cells or BMMs were detected by the Cell Counting Kit‐8 (CCK‐8, E‐CK‐A362, Elabscience) assay. Briefly, osteoclast precursor cells at a density of 1.5 × 10^5^/well were cultured in 96‐well plates and stimulated with different concentrations of Ski (30–45 μM) in a complete medium containing M‐CSF (10 ng/mL) with or without RANKL (30 ng/mL) for 24 h. After treatment, a 10 μL volume of CCK‐8 solution was added to each well and incubated at 37°C for 2 h, followed by optical density (OD) measurement via a spectrophotometer (Multiskan GO 1510, ThermoFisher Scientific) at a wavelength of 450 nm.

### 2.7 Tartrate‐Resistant Alkaline Phosphatase (TRAP) Staining

BMMs were cultured in 24 well plates at a density of 2 × 10^5^ cells/mL. After mature osteoclasts were differentiated from BMMs according to our previous study [9], the cells were washed three times with PBS and subsequently fixed with 4% paraformaldehyde (PFA) for 20 min at room temperature. TRAP staining was performed as previously described with slight modifications [36]. The matured osteoclasts were subjected to TRAP staining for 1 h and 50 min at 37°C. Deionised water was used throughout the cleaning process. Multi‐nuclei (≥ 3) TRAP‐positive cells were identified as osteoclasts and counted under a light microscope (AC 100‐240 V, Nexcope).

### 2.8 F‐Actin Staining

The cells were scoured 3 times with PBS and then subjected to 4% PFA for a 20 min fixation. Thereafter, cellular F‐actin rings were stained by phalloidin‐iflour 594 (ab176757, Abcam) probe at 37°C for 2 h, followed by 5 min of DAPI incubation to counter‐stain the nuclei. After 3 times PBS washes, the number and structure changes of F‐actin rings were observed by a fluorescence microscope (EVOS M7000) and analysed by ImageJ software. The F‐actin ring ratio was quantitatively analysed by calculating the number of osteoclasts with complete F‐actin rings divided by the overall number of osteoclasts.

### 2.9 Acridine Orange (AO) Staining

After cultured with M‐CSF and RANKL for 5 days, the matured osteoclasts were subjected to acridine orange (AO, A6014, Sigma‐Aldrich) solution in order to determine the cellular acid production as previously performed [9]. Cells were incubated in α‐MEM containing 10 μg/mL of AO solution for 15 min at 37°C and then subjected to 2 times α‐MEM washes. The acid vesicles of osteoclasts were visualised by a fluorescence microscope (Axio Observer 3, CarlZeiss). Each fluorescence intensity was expressed by the ratio of red to green light and measured by ImageJ (1.53c, National Institutes of Health, United States).

### 2.10 Measurement of Intracellular Calcium Oscillations

Calcium oscillations were investigated similarly to our previous study. The fluorescent probe Fluo‐4 was dissolved with a concentration of 20% F127 solution using DMSO as the solvent. ISO preparation: ddH2O as solvent, 105 mM NaCl, 5 mM KCl, 6 mM HEPES acid, 4 mM Na HEPES, 5 mM NaHCO_3_, 60 mM D‐Mannitol, 5 mM D‐glucose, 1.3 mM CaCl_2_ and 0.5 mM MgCl_2_. Briefly, BMMs were cultured in a cell culture dish (35 mm × 10 mm style) containing M‐CSF (10 ng/mL) at a density of 2.5 × 10^5^ cells/mL for 72 h and then changed into the OIM with or without Ski for 48 h. Subsequently, cells were incubated with 10% FBS supplemented with 5 μM Fluo‐4 probe solution for 50 min at 37°C in the dark and then washed once in ISO. Images were captured every 5 s for 20 min through a fluorescence microscope (EVOS M7000) and analysed by ImageJ software.

### 2.11 Western Blotting (WB) Assay

The experiments of WB were carried out as mentioned previously [9]. The cells were collected and lysed in pre‐cold radioimmunoprecipitation buffer (RIPA) buffer (R0020, Solarbio) containing 1% phosphatase (CW2383, Cwbio) and protease inhibitors (CW2200, Cwbio). The whole cell protein was extracted from cell lysates by centrifugation, and the protein concentrations were determined by a commercial BCA Protein Assay Kit (PC0020, Solarbio). Equal 20 μg of proteins from each sample were loaded into each lane and separated on 10% SDS‐PAGE gels by electrophoresis under denaturing conditions, then transferred to a PVDF membrane (Merck Millipore, ISEQ00010 0.2 μm). After 30 min of 5% solution of skimmed milk blocking, the membranes were incubated with primary antibodies against indicated proteins at 4°C overnight. Followed by 1.5 h incubation of secondary antibodies, the protein signals were detected by chemiluminescent HRP substrate (WBKLS0500, Merck Millipore) and observed via ChemiDoc touch imaging system (Bio‐Rad, CA, USA). Quantitative analyses of WB were performed by ImageJ software as previously described.

### 2.12 Reverse Transcription and Quantitative Polymerase Chain Reaction (RT‐qPCR)

Total RNA was extracted from cells using the RNAiso Plus (9109, Takara) method per the manufacturer's instructions. The reverse transcription (RT) reaction was performed on total RNA to synthesise cDNA via PrimeScript RT reagent Kits (RR047A, Takara). The cDNA was then amplified using SYBR Green qPCR Master Mix (AG11701, Accurate Biology) and RT‐qPCR was performed by a LightCycler 480II (Roche) to determine mRNA expression on a Roche Light Cycler 480II (Roche, Basel, Switzerland). GAPDH was employed as an endogenous control. All PCR reactions were performed in triplicate, and relative gene expressions were calculated using the $2^{-\Delta \Delta C_{t}}$ method. All primer sequences used in this study were listed in Table 1.

**TABLE 1:** Primer sequences used for qPCR.

Target	Primers (5′‐3′)
Traf6	F: AAAGCGAGAGATTCTTTCCCTG
	R: ACTGGGGACAATTCACTAGAGC
Nfatc1	F: CCGTTGCTTCCAGAAAATAACA
	R: TGTGGGATGTGAACTCGGAA
Ctsk	F: CTTCCAATACGTGCAGCAGA
	R: TCTTCAGGGCTTTCTCGTTC
Atp6v0d2	F: AACTCAGCAGGACTATGTCAACC
	R: CTTCTTCCTCATCTCCGTGTCAAT
MMP9	F: GCCCTGGAACTCACACGACA
	R: TTGGAAACTCACACGCCAGAAG
GAPDH	F: ACTTTGTCAAGCTCATTTCC
	R: TGCAGCGAACTTTATTGATG

### 2.13 Micro‐CT Scanning

Femurs were extracted after careful soft tissue removal and fixed in 4% PFA for 24 h, followed by rinsing and storage in 70% ethanol. MicroCT scanning (PerkinElmer Quantum GX2) of the distal femurs was performed at 70 kV and 120 μA. The region of interest (ROI) for analysis was defined as approximately 10–200 slices (2 mm) above the distal femur growth plate's highest point. Using the CT Analyser 12.0 program, the following parameters were assessed: bone volume fraction (BV/TV), bone surface density (BS/TV), trabecular number (Tb.N), trabecular separation (Tb.Sp) and trabecular thickness (Tb.Th). Additionally, three‐dimensional (3D) images were generated for comprehensive visualisation of bone structure.

### 2.14 Statistical Analysis

All quantitative data were shown as means ± standard deviations (SD). Two‐tailed Student's *t* test was used to compare the differences between the two independent groups; one‐way ANOVA followed by Sidak's multiple comparison test was used for grouped samples. A value of *p* < 0.05 was considered statistically significant. All statistical analyses were performed using GraphPad Prism 9 Software.

## 3 Results

### 3.1 The Prediction of Biological Pathway of Ski in Osteoporosis Prevention and Treatment

To explore the molecular mechanism by which Ski prevents and treats osteoporosis, a network pharmacology analysis was performed based on the molecular structure of Ski (Figure 1A). 59 putative Ski targets with a relevance > 0 were identified using the Swiss Target Prediction database, and 5936 human genes associated with osteoporosis were collected from the GeneCards database. Thereafter, mapping analysis between predicted targets of Ski and causative genes associated with osteoporosis revealed 44 overlapping therapeutic targets of Ski for osteoporosis prevention and treatment (Figure 1B).

To elucidate how these 44 overlapping genes exert therapeutic effects on osteoporosis, functional enrichment analysis was conducted using DAVID Bioinformatics Resources 6.8, and the enriched GO and KEGG pathways indicated that ‘Cellular calcium ion homeostasis’ and the ‘Calcium signalling pathway’ terms were highly ranked (Figure 1C–F). The results suggest these overlapping genes primarily involve in the regulations of cellular calcium signalling pathway via cellular calcium homeostasis in the plasma membrane and cytoplasm. These findings support Ski's potential therapeutic role in preventing and treating osteoporosis by modulating osteoclast differentiation and function via calcium signalling pathway.

### 3.2 Pharmacokinetic Visualisation of Ski's Bioavailability: Combined Radar Plot and BOILED‐Egg Analysis for Blood–Brain Barrier and Gastrointestinal Absorption Prediction

**FIGURE 2:**
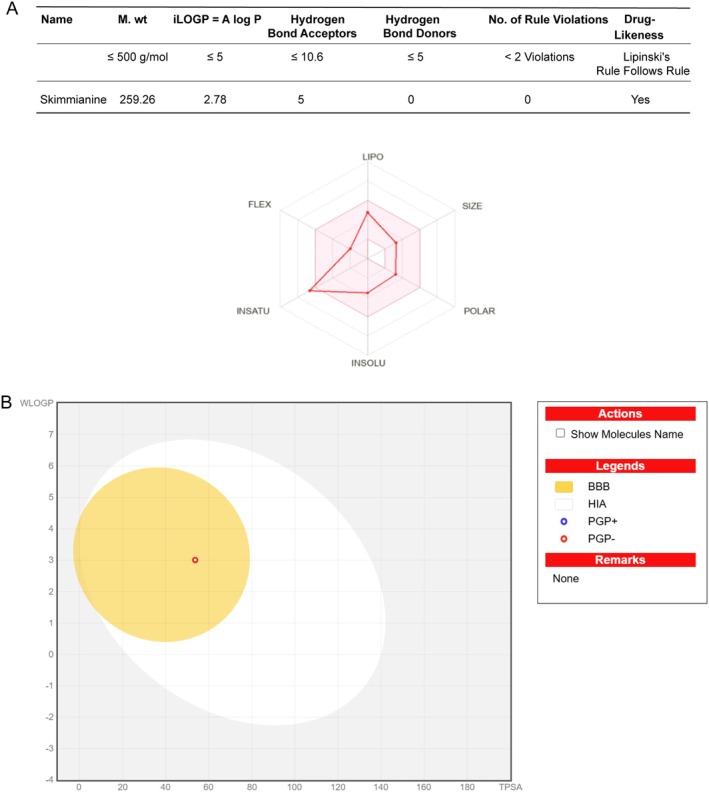
Pharmacokinetic visualisation of Ski's bioavailability: Combined Radar plot and BOILED‐Egg analysis for blood–brain barrier and gastrointestinal absorption prediction. (A) Radar analysis quantified six molecular descriptors: SIZE (molecular weight: 150–500 g/mol), LIPO (XLOGP3: −0.7 to *p* 5), POLAR (TPSA: 20–140 Å^2^), INSOLU (Log *S* (ESOL): 0–6), INSATU (sp^3^ carbon ratio: 0.25–1) and FLEX (rotatable bond count: 0–9), with optimal oral bioavailability indicated by the pink zone‌. (B) SwissADME‐derived BOILED‐Egg model (WLOGP = 3.01, TPSA = 53.72 Å^2^) positioned Ski in the yolk (BBB permeable) and white (GI absorbable) regions, reflecting dual absorption potential.

To evaluate Ski's potential as a drug candidate, its physicochemical parameters were analysed using Lipinski's rule of five and ADME methods [37]. As shown in Figure 2A, Ski satisfied Lipinski's criteria, indicating suitability for initial screening as an oral drug candidate. However, the radar plot bioavailability suggested incomplete oral bioavailability, with one parameter falling outside the optimal (pink) region. The BOILED‐Egg model, which predicts gastrointestinal absorption and brain penetration, positioned Ski within the ‘yolk’ area (Figure 2B), indicating predicted brain penetration. As illustrated in Figure 2B, Ski exhibited a WLOGP of 3.01 and TPSA of 53.72 Å^2^. Additionally, Ski was classified as a non‐substrate of P‐glycoproteins (PGP), denoted by a red dot. All calculated parameters fell within acceptable thresholds (WLOGP < 5.88 and TPSA < 131.6 A2). Collectively, these results suggest Ski has potential for favourable oral bioavailability, low predicted toxicity and efficient absorption rate.

### 3.3 Ski Maintains Bioactivity in BMMs and Impedes RANKL‐Induced Osteoclastogenesis

**FIGURE 3:**
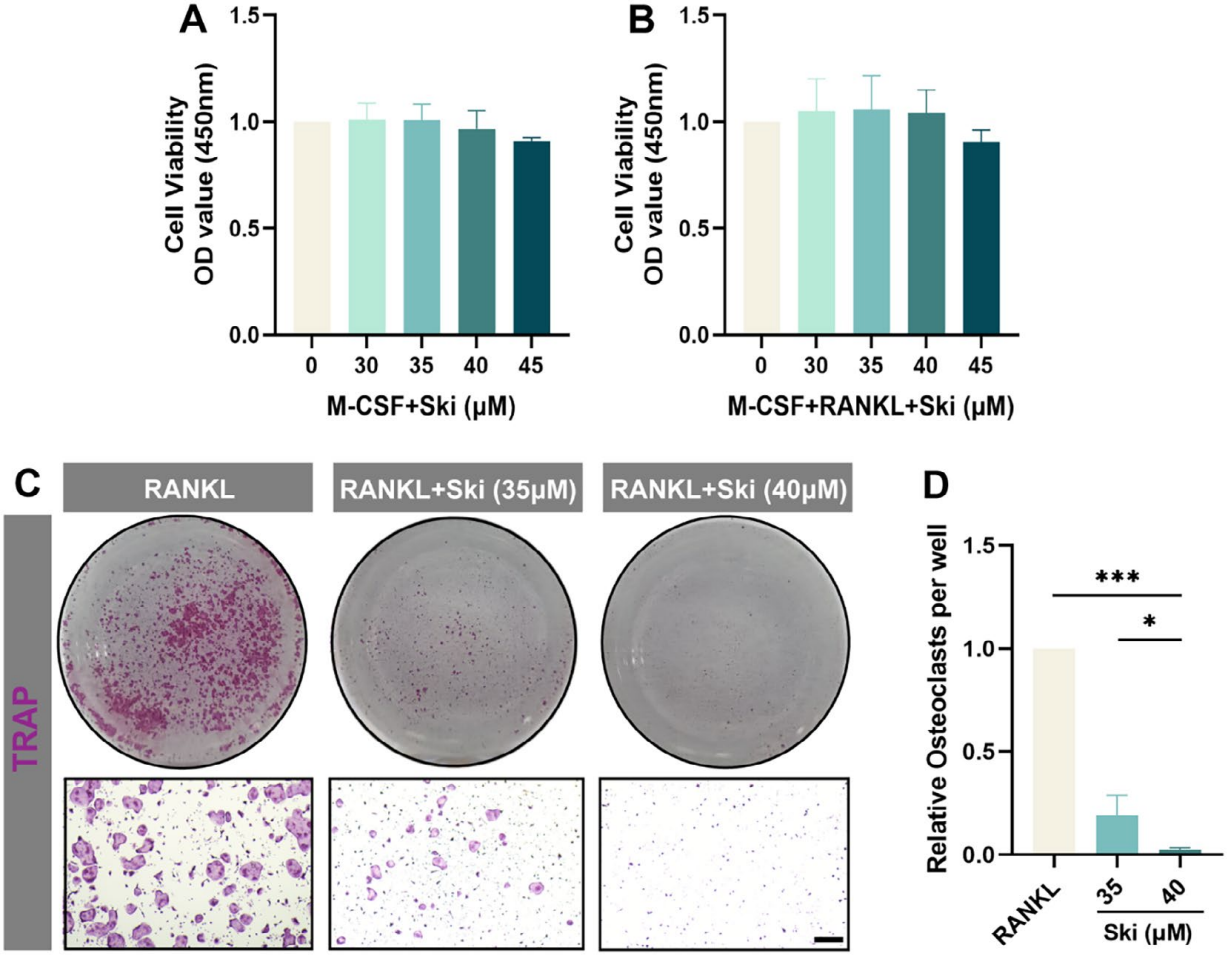
Ski maintains bioactivity in BMMs and impedes RANKL‐induced osteoclastogenesis. (A) CCK‐8 assay for cytotoxicity test. Bone marrow‐derived monocytes (BMMs) were treated with various concentrations of Ski (0–45 μM) stimuli in proliferating medium containing M‐CSF (10 ng/mL) only or in (B) osteoclast differentiation medium including M‐CSF and RANKL (30 ng/mL) for 24 h (*n* = 3). (C) TRAP staining of osteoclasts derived from BMMs under different catalyses. BMMs were induced with M‐CSF (10 ng/mL) and RANKL (30 ng/mL) for a 5‐day period under Ski (0, 35 and 40 μM) treatment. Scale bar = 200 μm. (D) Quantitative analysis of TRAP‐positive cells (containing more than three nuclei) (*n* = 3). Data were presented as mean ± SD. *p < 0.05; ***p < 0.001.

To confirm the predicted impact of Ski on osteoclast differentiation and function during osteoporosis as speculated, various assays were performed accordingly. The CCK8 results displayed the cell viability of BMMs remained unaffected by Ski concentrations up to 45 μM, regardless of the presence of M‐CSF or RANKL. No statistically significant cytotoxicity was observed below 45 μM, indicating Ski can be safely used on BMMs at concentrations below 45 μM (Figure 3A,B). Further, to establish a baseline for assessing cellular function effects in subsequent experiments, an intermediate concentration (40 μM) was selected. TRAP staining revealed that Ski, at non‐cytotoxic concentrations (0, 35 and 40 μM), remarkably ameliorated osteoclast formation in a concentration‐dependent manner (Figure 3C,D). Consequently, given the results from the CCK‐8 assay and TRAP staining, 40 μM Ski was chosen for further experiments. Compared to the control group, the addition of Ski (35 and 40 μM) apparently impeded osteoclast formation, confirming its potent inhibitory effect on RANKL‐stimulated osteoclastogenesis.

### 3.4 Ski Imposes Restrictions on Resorptive Function of Osteoclasts

**FIGURE 4:**
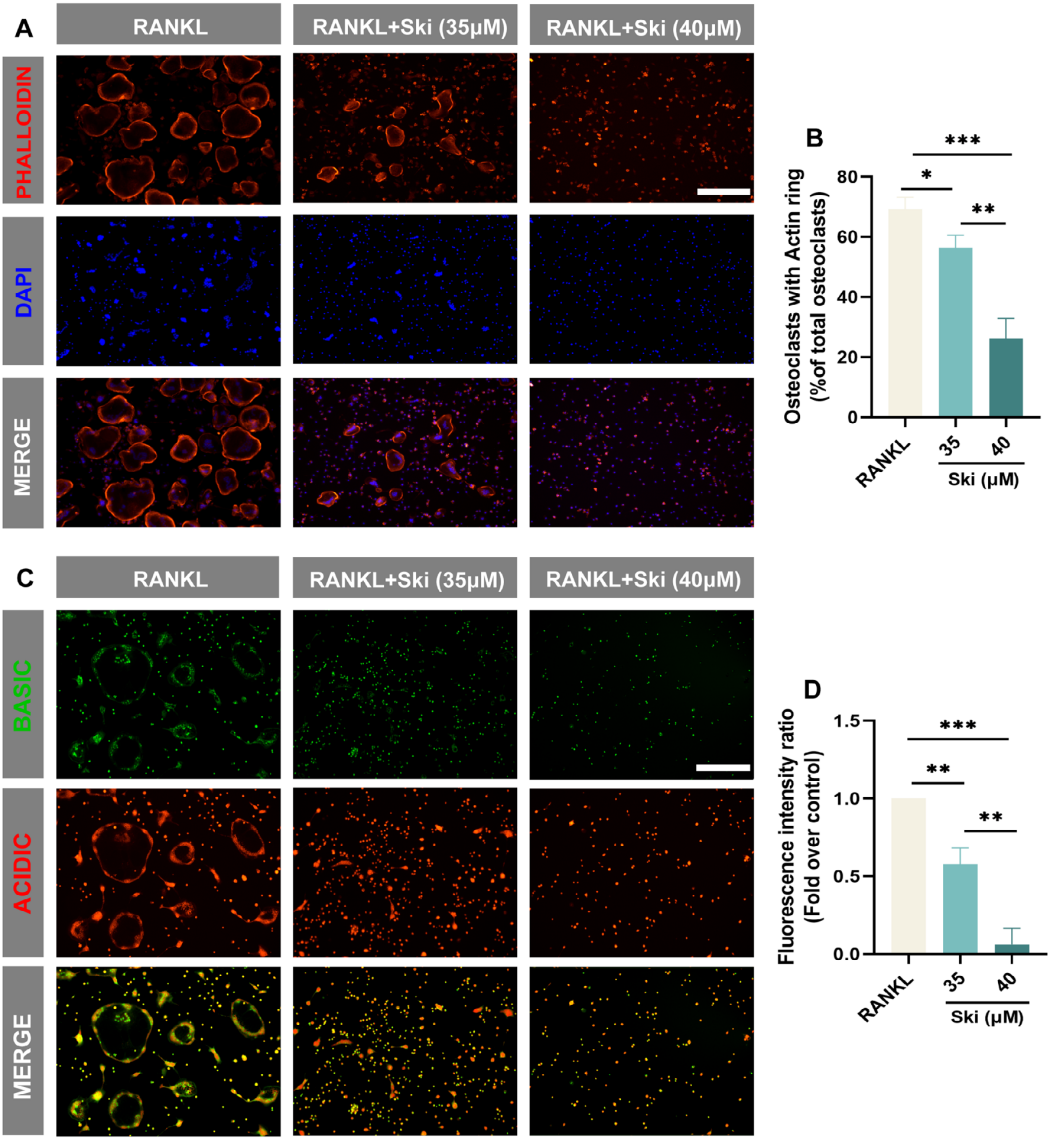
Ski imposes restrictions on resorptive function of osteoclasts. (A) F‐Actin ring staining. BMMs were induced with M‐CSF (10 ng/mL) and RANKL (30 ng/mL) for a 5‐day differentiation with or without Ski (35 and 40 μM) treatment. The F‐Actin rings (red) and nuclei (blue) of RANKL and Ski‐treated groups were stained using phalloidin‐ifour 594 and DAPI and observed under a fluorescence microscope. Scale bar = 300 μm. (B) The bar graph showed the proportion of osteoclasts with F‐Actin rings (*n* = 3). Data were presented as mean ± SD. *p < 0.05, **p < 0.01 and ***p < 0.001. (C) Acridine orange (AO) staining. BMMs were induced with M‐CSF (10 ng/mL) and RANKL (30 ng/mL) for a 4‐day differentiation with or without Ski (35 and 40 μM) treatment. The function of acid secretion in osteoclasts of acidic vesicles stained with AO solution was exhibited through the fluorescence microscope images. Scale bar = 300 μm. (D) Each fluorescence intensity was expressed by the ratio of red to green light and measured by ImageJ software (1.53c, National Institutes of Health, United States) (*n* = 3). Data were presented as mean ± SD. **p < 0.01. ***p < 0.001.

We next verify the impact of Ski on osteoclastic bone resorption, specifically assessing prerequisites including F‐actin ring formation and osteoclastic acidification. Phalloidin‐iflour 594 staining revealed that more than 69% of osteoclasts in the control group possessed intact F‐actin rings. In contrast, under Ski treatment (35 and 40 μM), the percentage of osteoclasts with intact rings substantially shrunk to 56% and 26%, respectively (Figure 4A,B). Meanwhile, AO staining showed reduced red fluorescence in osteoclasts after Ski addition, reflecting decreased accumulation of acidified compartments in Ski‐treated osteoclasts (Figure 4C,D). Collectively, these findings demonstrate that Ski mitigates the resorptive function of RANKL‐stimulated osteoclasts.

### 3.5 Ski Impedes the Expression of Osteoclast‐Specific Marker Genes

**FIGURE 5:**
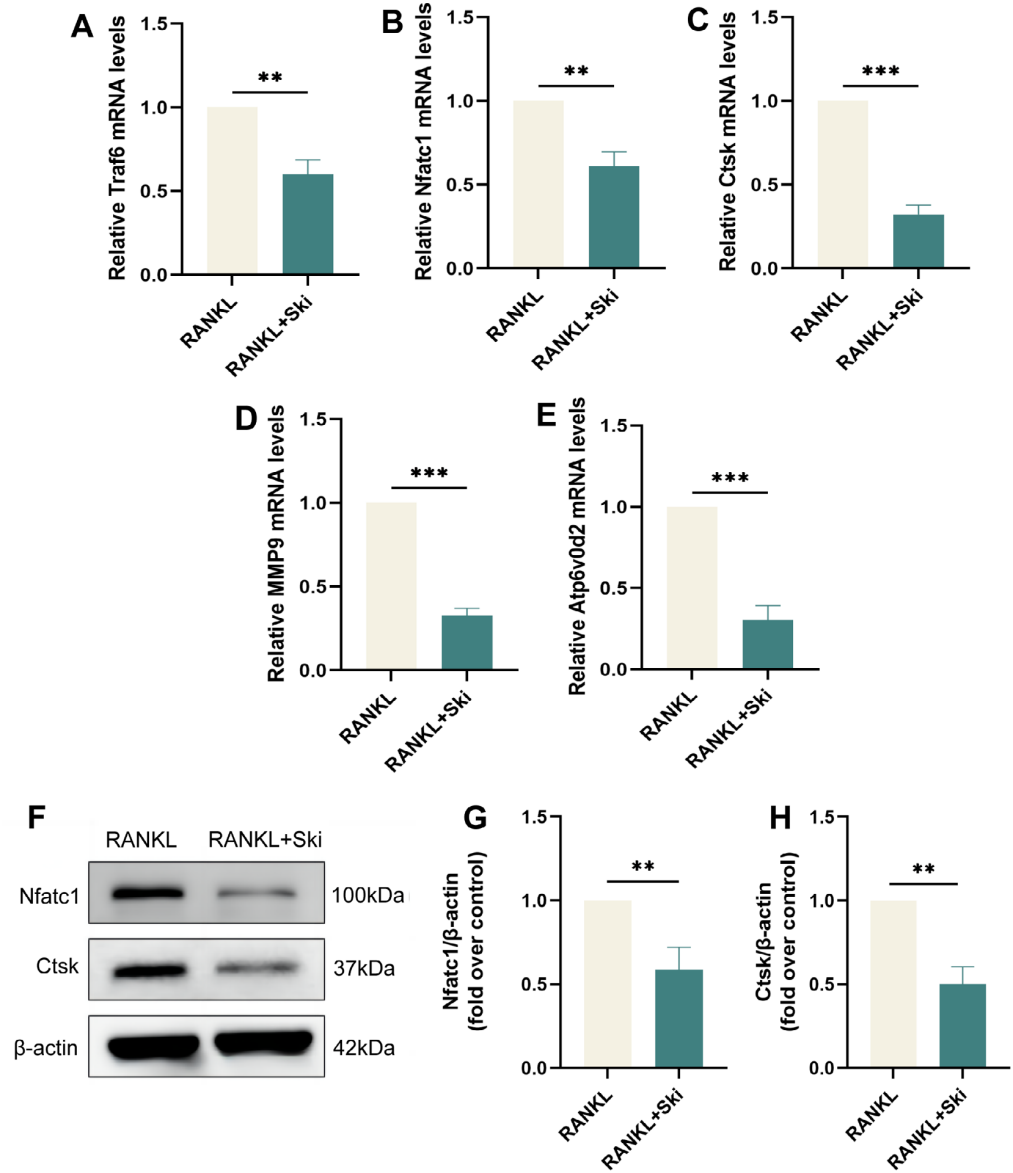
Ski impedes the expression of osteoclast‐specific marker genes. (A–E) RT‐qPCR illustrated the mRNA levels of Traf6 (A), Nfatc1 (B), Ctsk (C) MMP9 (D) and Atp6v0d2 (E) in RANKL and Ski (40 μM) groups. Quantitative results were normalised to GAPDH and presented as means ± SD (*n* = 3), **p < 0.01 and ***p < 0.001. (F–H) WB showed the protein level of Nfatc1 and Ctsk in RANKL and Ski (40 μM) groups. Quantitative results were normalised to β‐Actin and presented as means ± SD (*n* = 3). **p < 0.01.

To gain deeper insights into the role of Ski in osteoclast differentiation and function, we examined changes in RNA and protein levels of osteoclast‐specific markers. RT‐qPCR results revealed that the Ski‐treated group exhibited apparent reductions in the expression of osteoclast marker genes linked to differentiation (Traf6, NFATc1) and function (Ctsk, MMP9 and Atp6v0d2) compared to the RANKL‐treated group (Figure 5A–E). These findings were further validated by WB analyses, supporting the inhibitory effect of Ski on RANKL‐induced osteoclast differentiation and function at the molecular level (Figure 5F–H). In summary, these results illustrated that Ski eminently suppresses RANKL‐induced osteoclast differentiation and function.

### 3.6 Ski Negatively Affects Calcium Oscillations/Calcineurin/Nfatc1 Signalling During Osteoclastogenesis via the Suppression of ERp57 Expression

**FIGURE 6:**
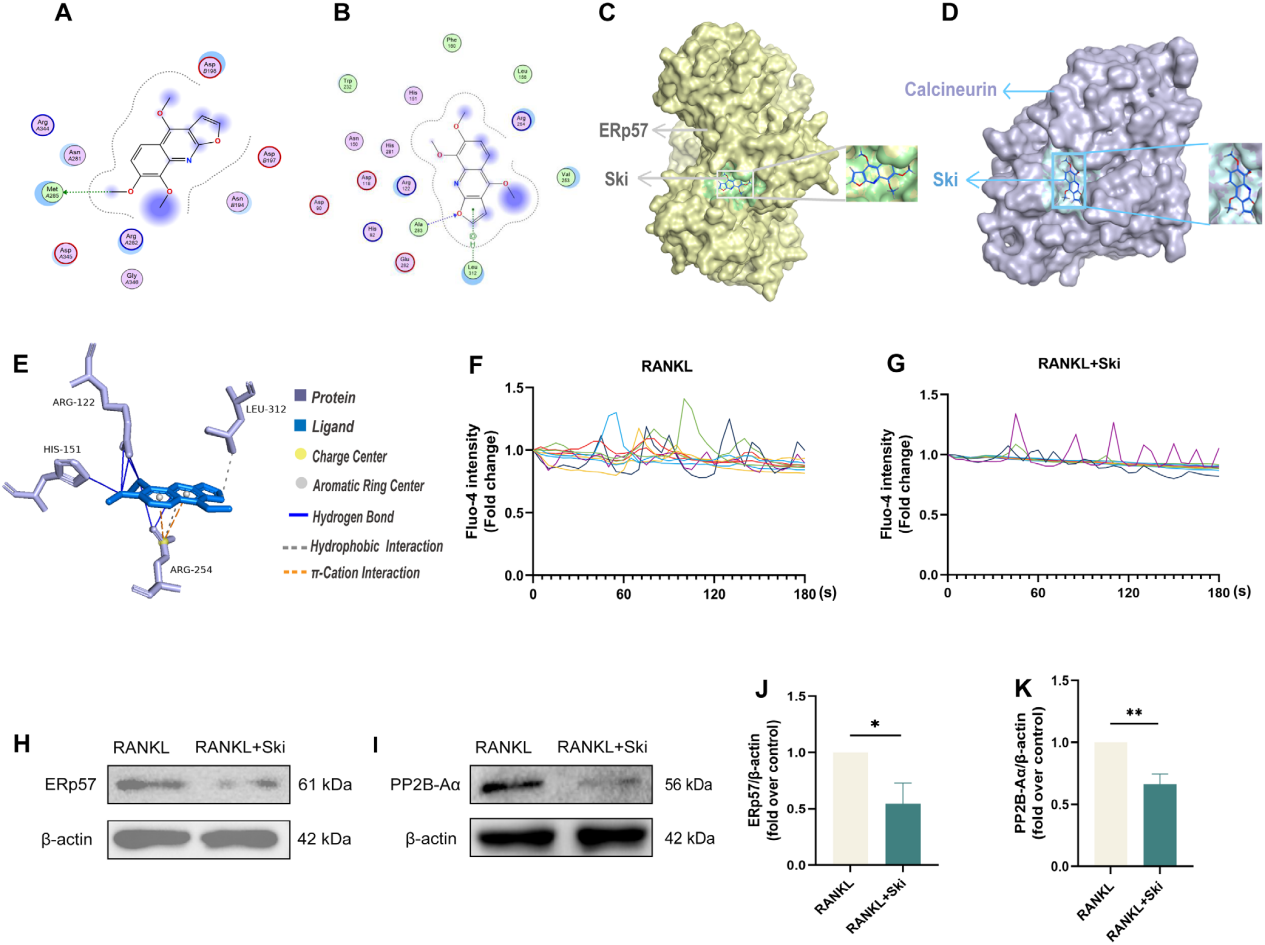
Ski negatively affects calcium oscillations/calcineurin/Nfatc1 signalling during osteoclastogenesis via the suppression of ERp57 expression. (A–D) Molecular docking ichnography and 3D molecular model between Ski and ERp57 (C), calcineurin (D). (E) Protein‐ligand interactions between Ski (blue) and calcineurin (violet, only interacting protein residues are shown). Interactions are drawn as coloured solid or dashed lines (mazarine, grey and orange). BMMs were induced with M‐CSF (10 ng/mL) and RANKL (30 ng/mL) with or without Ski (40 μM) for 48 h. 10 cells were selected from each treatment group (F, G) and indicated intensity of calcium oscillations. Images were captured every 5 s for 20 min through a fluorescence microscope (EVOS M7000). Quantitative analysis of fluorescence intensity was measured by ImageJ software. (H, J) WB showed the protein level of ERp57 in RANKL and Ski groups. (I, K) WB showed the protein level of PP2B‐Aα in RANKL and Ski groups. Quantitative results were normalised to β‐Actin and presented as means ± SD (*n* = 3). *p < 0.05, **p < 0.01.

Given the established role of ERp57‐mediated calcium signalling as confirmed by our previous study [38], and the critical function of calcium oscillations as upstream modulators in RANKL‐triggered calcineurin and osteoclast formation [37], we investigate whether Ski's suppressive effects on marker genes and cellular activities stem from its influence on calcium oscillations [39]. Molecular docking revealed potential binding sites between Ski and both ERp57 and calcineurin proteins (Figure 6A–D). Subsequently, analysis of ligand‐receptor interactions showed Ski‐calcineurin complex interactions (Figure 6E and Figure S1): two hydrophobic interactions were identified between two residues (ARG254, LEU312) and the Ski ligand. The number of hydrogen bonds formed was seven, and the residues involved in hydrogen bonds were ARG122, HIS151 and ARG254. The shortest distance between hydrogen bonds was 2.96 and the longest was 3.87 Å. Additionally, two π‐cation interactions were identified, implicating the residues of ARG254. As shown in (Figure 6F,G), calcium imaging demonstrated sustained, high‐magnitude oscillations in the RANKL‐treated group, contrasting sharply with significantly reduced calcium activity in the Ski‐treated group. Consistent with these findings, Ski treatment downregulated the expression of ERp57 protein (Figure 6H,J) and suppressed RANKL‐induced expression of PP2B‐Aα protein, a catalytic subunit of calcineurin (Figure 6I,K). Taken together, these results established that Ski attenuated osteoclastogenesis by inhibiting the ERp57‐mediated calcium oscillations/calcineurin/Nfatc1 signalling pathway.

### 3.7 Ski Attenuates Ovariectomy (OVX)‐Induced Bone Loss in Mice

**FIGURE 7:**
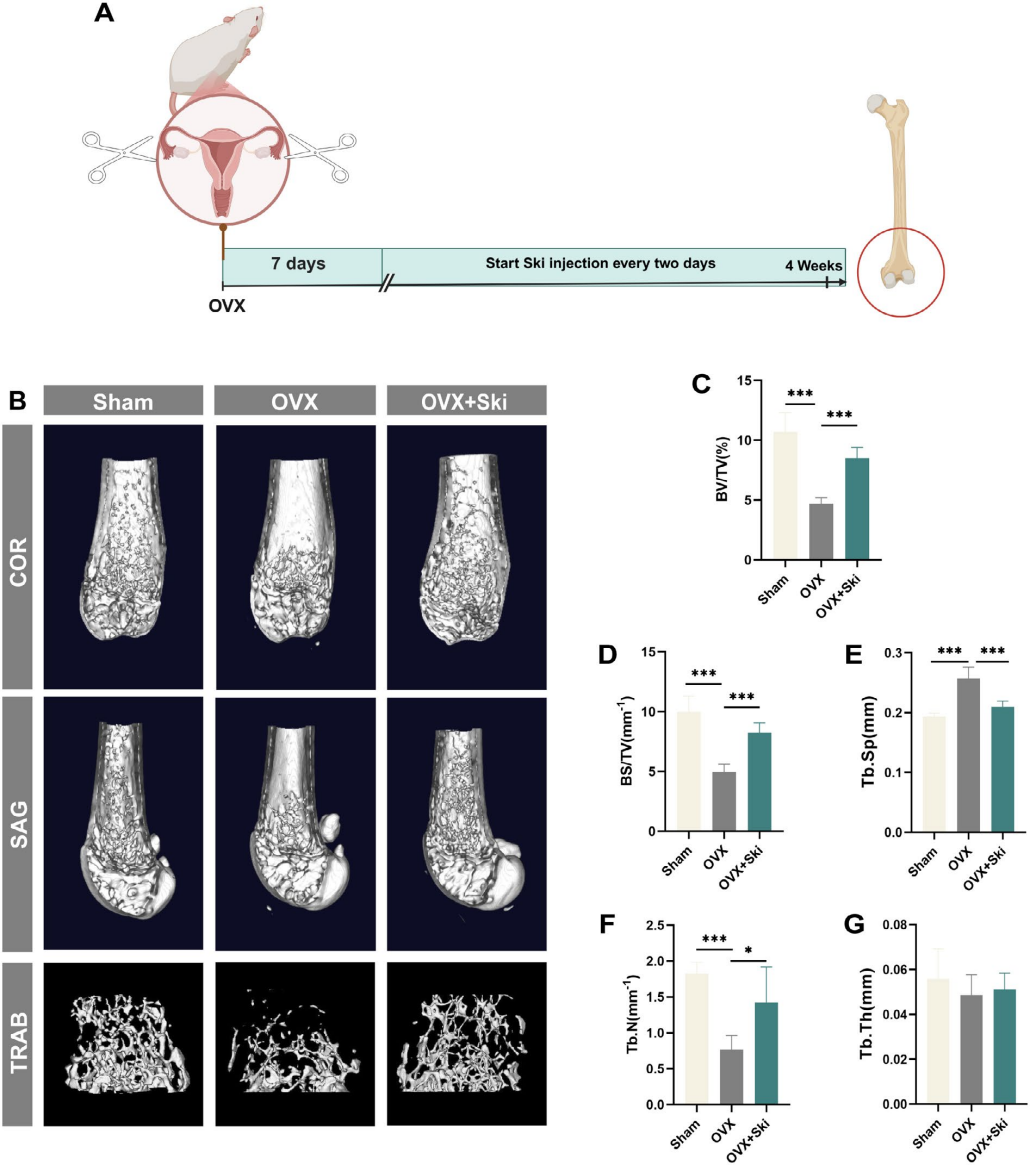
Ski attenuates ovariectomy (OVX)‐induced bone loss in mice. (A) ‌Flowchart delineating the in vivo pharmacological outcomes of Ski administration. (B) Three‐dimensional visualisation of femur distal regions demonstrates structural variations among Sham, OVX and Ski‐supplemented (30 mg/kg) intervention groups. (C–G) ‌Quantitative characterisation of trabecular morphometric indices in femoral bone revealed significant variations in: (C) bone volume fraction (BV/TV), (D) bone surface density (BS/TV), (E) trabecular separation (Tb.Sp), (F) trabecular number (Tb.N) and (G) trabecular thickness (Tb.Th). Data were presented as mean ± SD (*n* = 5 per group, *p < 0.05, ***p < 0.001).

To validate Ski's therapeutic potential, we conducted in vivo studies using established OVX mouse models, building upon prior network pharmacology, pharmacokinetic analyses and in vitro experiments (Figure 7A). From postoperative weeks 1–4, OVX + Ski mice received intraperitoneal Ski injections (30 mg/kg) every 2 days, while sham‐operated and OVX control groups received isotonic saline via identical procedures. Three‐dimensional micro‐CT reconstructions revealed compromised trabecular integrity in OVX mice, whereas Ski treatment preserved bone microarchitecture by maintaining trabecular node‐to‐node connectivity (Figure 7B). Relative to sham controls, the OVX mice exhibited marked reductions in bone volume fraction (BV/TV), bone surface density (BS/TV) and trabecular number (Tb.N), along with increased trabecular separation (Tb.Sp).‌ Ski treatment effectively reversed these osteoporotic changes, elevating BV/TV, BS/TV and Tb.N while reducing Tb.Sp compared to untreated OVX mice (Figure 7C–F)‌. Notably, Tb.Th remained statistically comparable across all groups (Figure 7G). These findings collectively demonstrated Ski's therapeutic efficacy in mitigating OVX‐induced bone deterioration.

## 4 Discussion

**FIGURE 8:**
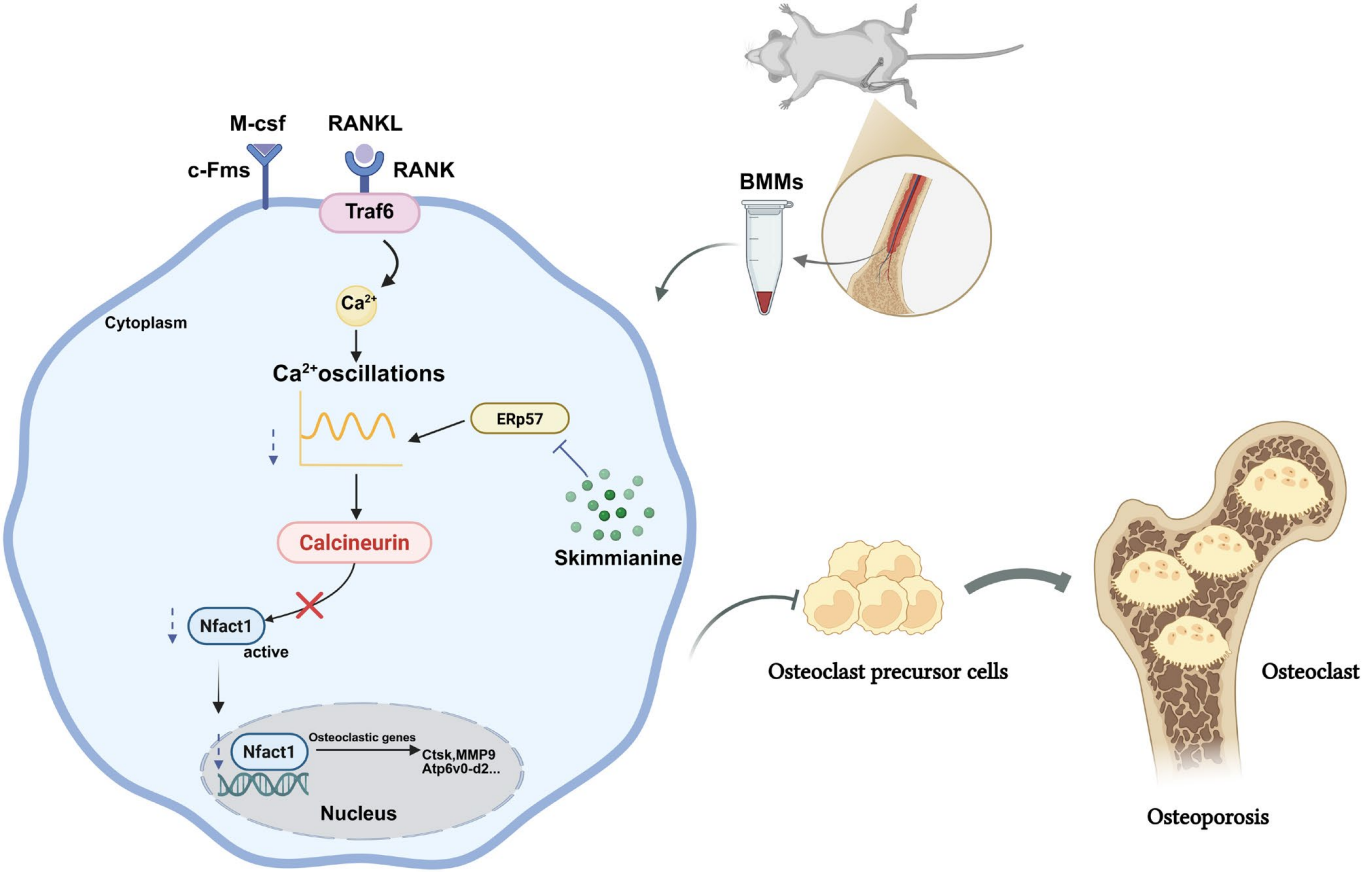
A working schematic diagram demonstrates Ski mitigates osteoclastogenesis through suppressing ERp57‐driven calcium oscillations–calcineurin–NFATc1 signalling. RANKL–RANK interaction initiates intracellular calcium oscillations, a signalling cascade that subsequently activates calcineurin‐dependent NFATc1 transcriptional activity. Ski inhibited ERp57‐driven intracellular calcium oscillations and the expression of calcineurin, which attenuated NFATc1 activation, subsequently leading to the reduction of osteoclast‐specific gene expression, including Ctsk, MMP9 and Atp6v0d2. RANK: TNF receptor superfamily member 11a; RANKL: Receptor activator of nuclear factor‐κB ligand; Traf6: TNF receptor‐associated factor 6; Nfatc1: Nuclear factor of activated T‐cells 1; Ctsk: Cathepsin K; MMP9: Matrix metalloproteinase 9; Atp6v0‐d2: ATPase H+ transporting V0 subunit d2.

Previous studies identified Ski, the most abundant furoquinoline alkaloid in Zanthoxylum genus, as an anti‐inflammatory agent. While numerous studies link suppression of calcium signalling pathways to reduced bone destruction in osteoporosis [40, 41], the specific role of Ski in osteoclast‐mediated osteoporosis remained unexplored. Through integrated network pharmacology, pharmacokinetic analyses and experimental validations, we demonstrate that Ski significantly inhibits osteoclast differentiation by suppressing the ERp57‐driven calcium oscillations/calcineurin/Nfatc1 signalling (Figure 8). This discovery offers novel therapeutic potential for postmenopausal osteoporosis.

This study provides the first verification of a specific association between Ski's targets and osteoporosis‐related pathogenic genes through integrated drug target prediction. From target prediction of Ski and GO/KEGG pathway analysis, our results revealed Ski may affect calcium signalling pathway via cellular calcium homeostasis that were carried out in the plasma membrane and cytoplasm [42, 43]. Excessive osteoclastogenesis is established as a primary driver of disordered bone remodelling in osteoporosis [12]. Therefore, targeting OC precursors, key mediators of bone loss, represents a crucial therapeutic strategy for osteoporosis prevention and treatment [44]. From a pharmacological perspective, ADME data modelling offers valuable insights into a drug's metabolic processes and absorption patterns [45]. Swiss ADME analysis showed five out of six radar plot parameters within the optimal bioavailability range, suggesting Ski's potential as an oral agent. Compliance with Lipinski's rule of five and bioavailability radar plots confirmed Ski's drug‐likeness and oral viability [46]. Based on these criteria, Ski exhibited a promising oral bioavailability profile for pharmaceutical development. Herein, our integrated network pharmacology and pharmacokinetic analyses identify Ski as a promising candidate for osteoporosis prevention and treatment.

We validated the actual impacts of Ski on osteoclast differentiation and function. It is well established that osteoclast formation is primarily mediated by RANKL‐RANK signalling, which also promotes the secretion of proteolytic enzymes, including Ctsk, MMP9 and Atp6v0d2 [47]. Matsuo et al. first described this Ski in 1904 as one of the new furoquinoline alkaloids from Skimmia japonica leaf and stem [48]. M. Ratheesh et al. reported the anti‐inflammatory properties of Ski [49]. In our study, CCK8 assays established a non‐cytotoxic concentration range for Ski (≤ 45 μM), enabling subsequent experiments. Notably, Ski significantly reduced both osteoclast numbers and osteolytic function even under RANKL stimulation (Figures 3 and 4). The osteolytic activity of osteoclasts relies on the presence of a sealing zone composed of an F‐actin column core (ring‐like structure) which attached tightly to the bone surface as well as the acidic microenvironment formed by acidic vesicles [50]. Pretreatment with increasing Ski concentrations dose‐dependently suppressed both F‐actin ring formation and acidified compartment development in RANKL‐induced osteoclasts. These cellular behaviours changes corresponded with down‐regulation of key regulators involved in osteoclastogenesis and bone resorption, including Traf6, Nfatc1, Ctsk, MMP9 and Atp6v0d2 [51].

Experimental evidences confirm that ERp57, a thiol‐oxidoreductase, modulates calcium oscillations which are essential for osteoclast development and function [38]. Calcium oscillations, defined by rhythmic fluctuations in intracellular calcium level, trigger signalling cascades critical for osteoclast biology [12, 13]. On the other hand, calcineurin, a calcium‐activated phosphatase central to calcium signalling, serves as the primary regulator of Nfatc1. This key transcription factor undergoes nuclear translocation and autoamplification upon calcium/calcineurin pathway activation [14, 52, 53]. Notably, calcium channel inhibitors block osteoclastogenesis [54, 55], confirming the calcineurin–Nfatc1 axis as essential for osteoclast differentiation through intracellular calcium elevation [56, 57]. Our study revealed significantly enhanced calcineurin expression in osteoclasts, which is in agreement with prior studies [41, 58]. While treated with Ski, we found that it could suppress ERp57‐mediated calcium oscillations, reduce calcineurin expression under RANKL stimulation, and block RANKL‐induced Nfatc1 nuclear translocation during osteoclast formation (Figure 6). In vivo, the Ski‐treated group exhibited significantly higher bone mass than untreated OVX mice (Figure 7), suggesting that Ski effectively mitigated bone loss in OVX mice. Collectively, Ski's suppression of the ERp57‐mediated calcium oscillations/calcineurin/Nfatc1 signalling potently inhibits osteoclast differentiation and function, thereby attenuating osteoporotic bone loss.

While the current findings provide robust evidence for Ski's role in suppressing osteoclast differentiation by the modulation of the calcium signalling pathway, future investigations are set to explore potential translational applications based on its favourable pharmacokinetic properties. The oral bioavailability of Ski and its anti‐osteoclast hyperactivation properties render it a promising candidate for development into an oral therapeutic for chronic bone loss disorders, or a component of the combination therapy with prevailing antiresorptive drugs. Building on these findings, employing genetic approaches involving ERp57 conditional knockout and transgenic overexpression models, we intend to elucidate the intricate molecular interaction between ERp57 and Ski, thereby establishing ERp57 as the main intermediary of Ski in the osteoclast lineage. This will enhance Ski's potential for clinical translation through formulation refinement and preclinical evaluation. Although pharmacokinetic predictions suggested Ski's good oral bioavailability (> 30%), actual plasma concentrations, metabolic stability and tissue accumulation require experimental validation. These aspects were beyond the scope of the current study but will be prioritised in follow‐up research. For instance, metabolite analysis will be performed using liquid chromatography–tandem mass spectrometry in animal models [59]. However, the Ski (40 μM) dose aligns with the range of IC50 values (> 30 μM) reported for Ski‐mediated inhibition in HEL cell lines [60], suggesting its biological plausibility despite pharmacokinetic uncertainties. Additionally, in future studies, 44 candidate genes obtained through network pharmacology also warrant investigation.

## 5 Conclusion

Conclusively, our study integrates network pharmacology, pharmacokinetic analyses and pharmacological validation to demonstrate that Ski, an active component derived from the Zanthoxylum genus, inhibits osteoclastogenesis in vitro. This effect occurs through the suppression of ERp57‐driven calcium oscillations/calcineurin/Nfatc1 signalling and subsequent osteoclast‐related marker gene expression. Our findings provide compelling evidence that Ski ameliorates osteoporotic bone loss and represents a promising therapeutic candidate, establishing a theoretical foundation for developing Ski‐based osteoporosis treatments.

## Author Contributions

**Yongshuang Lv:** data curation (equal), formal analysis (equal), investigation (equal), validation (equal), visualization (equal), writing – original draft (equal). **Xin Zhang:** data curation (equal), formal analysis (equal), investigation (equal), validation (equal), visualization (equal), writing – original draft (equal). **Qizhen Lu:** data curation (equal), formal analysis (equal), methodology (equal), validation (equal), visualization (equal). **Yi Zhou:** methodology (lead), validation (lead). **Weiyi Wang:** methodology (supporting), validation (supporting). **Maosheng Yang:** methodology (supporting), validation (supporting). **Tao Yuan:** methodology (supporting), validation (supporting). **Yikai Liu:** methodology (equal), validation (equal). **Shui Sun:** funding acquisition (equal), project administration (equal), supervision (equal), writing – review and editing (equal). **Ziqing Li:** funding acquisition (equal), project administration (equal), supervision (equal), writing – review and editing (equal).

## Ethics Statement

All experiments involving mice were performed following the protocol approved by the Institutional Animal Care and Use Committee (IACUC) of the Shandong Provincial Hospital Affiliated to Shandong First Medical University (Shandong, China; No. 2023‐034).

## Conflicts of Interest

The authors declare no conflicts of interest.

## Supporting information


**Figure S1:** Interaction maps between Ski (orange) and calcineurin (blue, only interacting protein residues are shown), and the comprehensive representation of interaction properties.

**Table S1:** Skimmianine (Ski), physicochemical and pharmacokinetic properties, ADME parameters, drug‐likeness, and medicinal chemistry properties predictions using SwissADME.

## Data Availability

The datasets used and/or analysed during the current study are available from the corresponding author on reasonable request.
