# Supplementary material for: Skimmianine Attenuates Osteoclast Activity by Suppressing ERp57‐Driven Calcium Oscillations/Calcineurin/Nfatc1 Signalling in Postmenopausal Osteoporosis
Source: J Cell Mol Med. 2025 Aug 12;29(15):e70777. doi: 10.1111/jcmm.70777 (PMC12343327; doi:10.1111/jcmm.70777)

**Supplementary Fig 1. Interaction maps between Ski (orange) and calcineurin (blue, only interacting protein residues are shown) , and the comprehensive representation of interaction properties.**


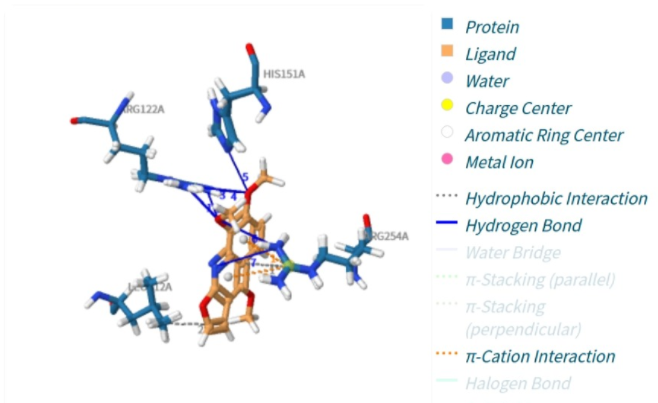


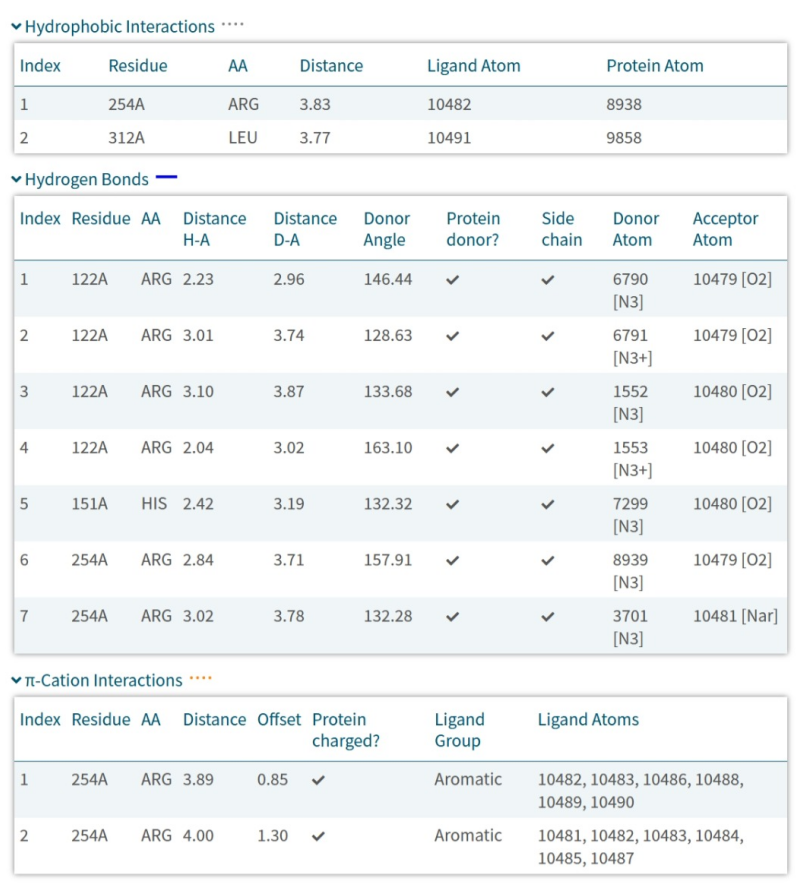

Supplement: Supplementary file 1 — Figure S1: Interaction maps between Ski (orange) and calcineurin (blue, only interacting protein residues are shown), and the comprehensive representation of interaction properties. [file JCMM-29-e70777-s001.docx]
