# Supplementary material for: Skimmianine Attenuates Osteoclast Activity by Suppressing ERp57‐Driven Calcium Oscillations/Calcineurin/Nfatc1 Signalling in Postmenopausal Osteoporosis
Source: J Cell Mol Med. 2025 Aug 12;29(15):e70777. doi: 10.1111/jcmm.70777 (PMC12343327; doi:10.1111/jcmm.70777)

**Supplementary Table 1. Skimmianine (Ski), physicochemical and pharmacokinetic properties, ADME parameters, drug-likeness, and medicinal chemistry properties predictions using SwissADME.**


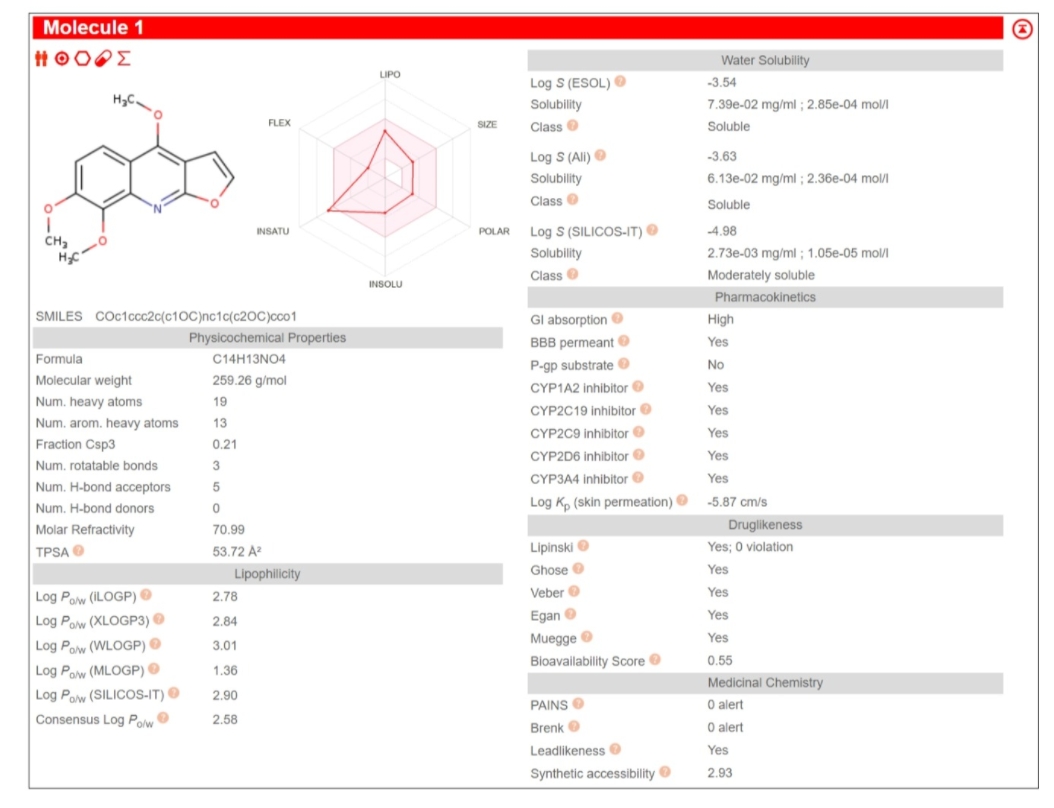

Supplement: Supplementary file 2 — Table S1: Skimmianine (Ski), physicochemical and pharmacokinetic properties, ADME parameters, drug‐likeness, and medicinal chemistry properties predictions using SwissADME. [file JCMM-29-e70777-s002.docx]
